# Shave removal for videogame controller–induced knuckle pads

**DOI:** 10.1002/ccr3.3850

**Published:** 2021-01-28

**Authors:** Kendall Flanigan, Steven Kent, Kathryn Anne Potter

**Affiliations:** ^1^ Department of Dermatology Medical College of Georgia Augusta GA USA

**Keywords:** knuckle pads, occupational dermatology, shave removal

## Abstract

Videogame controller–induced knuckle pads may present in a strikingly unique distribution. Successful paring without recurrence can be achieved when combined with removal of the original insult.

## Graphical abstract

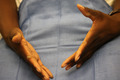

## 1 CLINICAL IMAGE

Knuckle pads are a benign and typically treatment‐resistant entity found on extensor surfaces of the fingers. We present a case of videogame controller–induced knuckle pads on the ulnar and radial surfaces of the digits. Successful treatment without recurrence was achieved when paring was combined with removal of the original insult.

**FIGURE 1:**
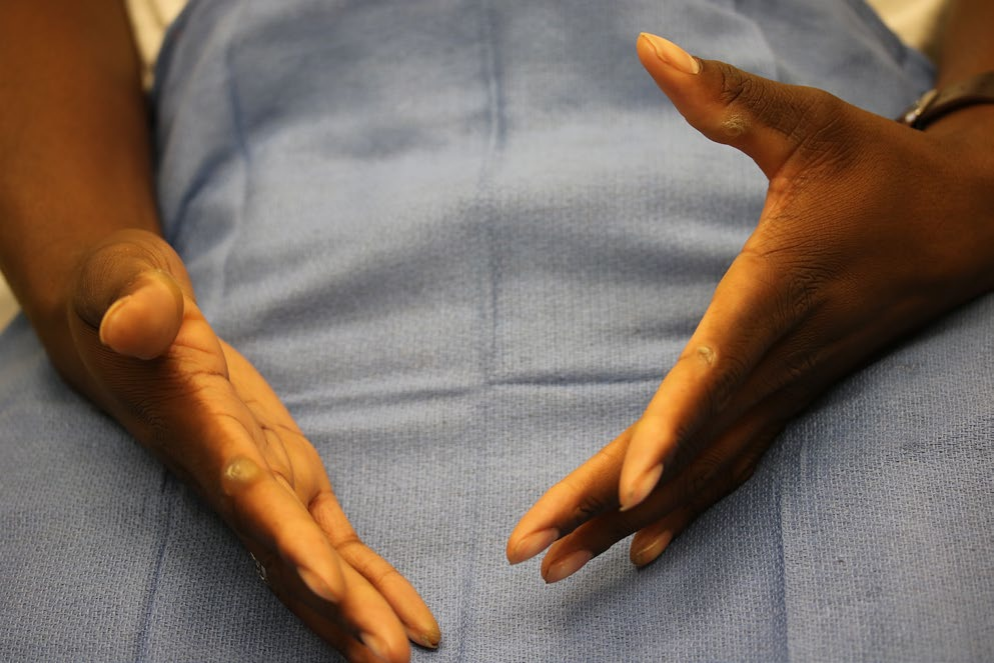
Videogame controller–induced knuckle pads on the ulnar surface of the proximal interphalangeal joint of the first digits and the radial surface the second digits bilaterally

A healthy 19‐year‐old male was referred to our clinic with concern for keratoderma given clinical diagnosis of knuckle pads. He presented with firm, nontender, hyperkeratotic papules on the ulnar surface of the proximal interphalangeal joints of the first digits and the radial surface of the second‐fourth digits bilaterally (Figure 1). These lesions had developed over three years and persisted despite cryosurgery. No family history of similar findings was noted. The patient and his mother believed them to be idiopathic. Upon examining the distribution, the patient was asked if he plays videogames. He played many hours per night, and the way he held the controller explained the striking distribution of the knuckle pads.

Knuckle pads are benign, hyperkeratotic papules, nodules, or plaques typically found on the extensor surfaces of the metacarpophalangeal or interphalangeal joints.^1^ They may be idiopathic, disease‐associated, or related to repetitive trauma.^1^ Frequent videogame use can result in trauma‐induced knuckle pads.^2^ Topical therapy and local destruction typically result in high recurrence rates.^1^

#15 blade paring of the lesions combined with physician‐directed behavioral modifications—limiting videogame controller use and taking frequent breaks—led to successful treatment (Figure 2). For trauma‐induced knuckle pads, we recommend paring and removing traumatic insults.

**FIGURE 2:**
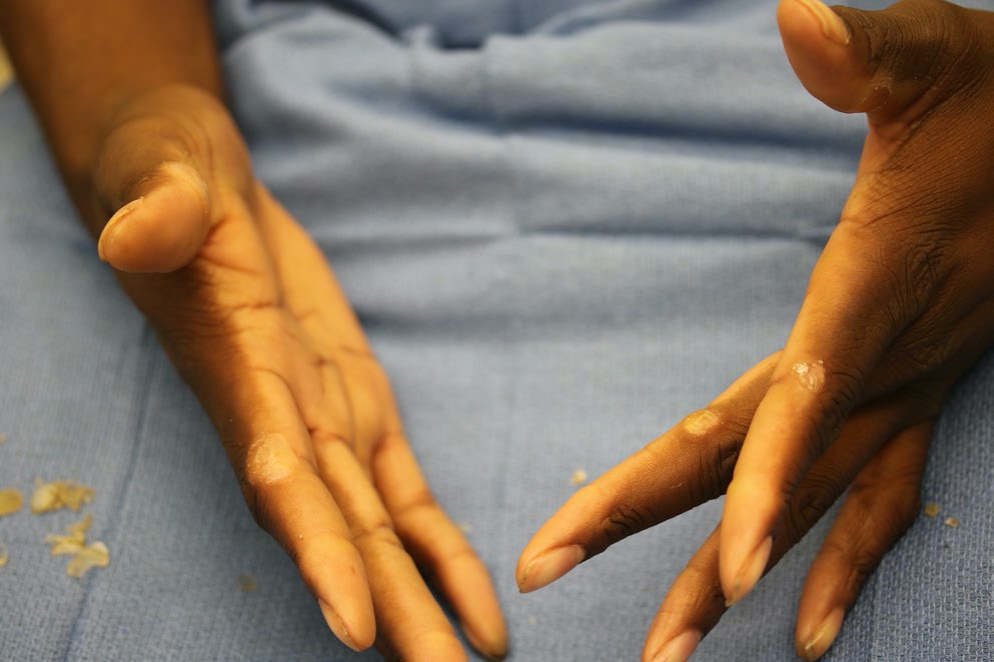
Videogame controller–induced knuckle pads, post paring with a #15 blade

## CONFLICT OF INTEREST

None declared.

## AUTHOR CONTRIBUTIONS

All authors: made substantial contributions to the preparation of this manuscript and approved the final version for submission.

KF: performed literature search, drafted initial version of manuscript, and revised manuscript. SK: contributed significant revisions to the manuscript and acquired images. KP: contributed significant revisions to the manuscript.

## ETHICS

Patient consent for publication available upon request.

## Data Availability

Data sharing is not applicable to this paper as no datasets were generated or analyzed.
